# Electrochemical Sensors Modified with Combinations of Sulfur Containing Phthalocyanines and Capped Gold Nanoparticles: A Study of the Influence of the Nature of the Interaction between Sensing Materials

**DOI:** 10.3390/nano9111506

**Published:** 2019-10-23

**Authors:** Ana Isabel Ruiz-Carmuega, Celia Garcia-Hernandez, Javier Ortiz, Cristina Garcia-Cabezon, Fernando Martin-Pedrosa, Ángela Sastre-Santos, Miguel Angel Rodríguez-Perez, Maria Luz Rodriguez-Mendez

**Affiliations:** 1 Group UVASENS, Escuela de Ingenierías Industriales, Universidad de Valladolid, Paseo del Cauce, 59, 47011 Valladolid, Spain; anaisabel.ruiz@uva.es (A.I.R.-C.); celiagarciahernandez@gmail.com (C.G.-H.); crigar@eii.uva.es (C.G.-C.); fmp@eii.uva.es (F.M.-P.); 2 BioecoUVA Research Institute, Universidad de Valladolid, 47011 Valladolid, Spain; marrod@fmc.uva.es; 3 Área de Química Orgánica, Instituto de Bioingeniería, Universidad Miguel Hernández de Elche, 03202 Elche, Spain; jortiz@umh.es (J.O.); asastre@umh.es (Á.S.-S.)

**Keywords:** electrochemical sensor, phthalocyanine, gold nanoparticle, catechol

## Abstract

Voltametric sensors formed by the combination of a sulfur-substituted zinc phthalocyanine (ZnPc^RS^) and gold nanoparticles capped with tetraoctylammonium bromide (AuNP^tOcBr^) have been developed. The influence of the nature of the interaction between both components in the response towards catechol has been evaluated. Electrodes modified with a mixture of nanoparticles and phthalocyanine (AuNP^tOcBr^/ZnPc^RS^) show an increase in the intensity of the peak associated with the reduction of catechol. Electrodes modified with a covalent adduct-both component are linked through a thioether bond-(AuNP^tOcBr^-S-ZnPc^R^), show an increase in the intensity of the oxidation peak. Voltammograms registered at increasing scan rates show that charge transfer coefficients are different in both types of electrodes confirming that the kinetics of the electrochemical reaction is influenced by the nature of the interaction between both electrocatalytic materials. The limits of detection attained are 0.9 × 10^−6^ mol∙L^−1^ for the electrode modified with the mixture AuNP^tOcBr^/ZnPc^RS^ and 1.3 × 10^−7^ mol∙L^−1^ for the electrode modified with the covalent adduct AuNP^tOcBr^-S-ZnPc^R^. These results indicate that the establishment of covalent bonds between nanoparticles and phthalocyanines can be a good strategy to obtain sensors with enhanced performance, improving the charge transfer rate and the detection limits of voltammetric sensors.

## 1. Introduction

Catechol is an important member of the family of phenols that can be found as an antioxidant in foods. Different types of electrodes have been described in the literature to assess the concentration of catechol in solution [1,2,3,4,5,6,7].

Phthalocyanines (Pcs) have attracted interest as chemical modifiers in electrochemical sensors dedicated to the detection of phenols due to their well-known electrocatalytic activity. Their electrochemical properties can be modified by introducing substituents in the aromatic ring [8,9,10,11,12,13]. Over the last decade, phthalocyanines have been linked covalently to a number of molecules, including fullerenes [14,15], perylenes [16,17], carbon-nanotubes [18,19], graphite, and nanoparticles [20,21,22,23].

The electrocatalytic properties of gold nanoparticles (AuNPs) are also well established [24,25,26,27], and a variety of uncapped and capped AuNPs have been successfully used to detect phenols [28,29,30].

One possible strategy to improve the performance of electrochemical sensors could be to develop composites formed by combinations of electrocatalytic materials, in order to generate synergistic effects [13,31,32]. Synergistic effects have been observed in AuNP/Pcs composites obtained by introduction weak interactions between both materials by means of mixing [32,33,34], self-assembling [35], the Langmuir-Blodgett (LB) [36,37], or electrodeposition techniques [38].

In spite of the interest in these combinations, the influence of the nature of the interaction between both components in the sensing properties remains largely unexplored.

The aim of this work is to develop new voltammetric sensors based on combinations of gold nanoparticles and sulfur-substituted zinc phthalocyanines and to analyze the electron transfer process, as well as the existence of synergistic effects between both components in the absence and presence of covalent links.

For this purpose, tetraoctylammonium bromide-gold nanoparticles (AuNP^tOcBr^) and 2-{2′-[(5″-Acetylthiopentyloxo)amino]ethoxy}-9(10),16(17),23(24)-tri-tert-butylphthalocyaninate Zn(II) (ZnPc^RS^) have been synthesized. These species have the appropriate substituents necessary to obtain a covalently linked adduct in which the nanoparticles and the phthalocyanines have been linked covalently through thiol bonds (AuNP^tOcBr^-S-ZnPc^R^).

The sensing properties towards catechol of an ITO substrate modified with the adduct, have been compared with the responses of an ITO glass covered with a mixture of both components (AuNP^tOcBr^/ZnPc^RS^). In addition, the response of a mixture formed by AuNP^tOcBr^ and a dimeric phthalocyanine where the sulfur groups are blocked AuNP^tOcBr^/ZnPc^R^-S-ZnPc^R^ have also been analyzed.

In all cases, studies at increasing scan rates have been carried out to evaluate the influence of the type of bond in the charge transfer rates. The limits of detection have also been calculated and compared.

## 2. Materials and Methods

Chemicals and solvents were of reagent grade (Aldrich Chemical Ltd., St. Louis, MO, USA). Reagents to prepare gold nanoparticles were: HAuCl4·xH2O (99.9%, min. 49% Au, Alfa Aesar, Haverhill, MA. USA), tetraoctylammonium bromide (98%, Aldrich Chemical. Ltd., St. Louis, MO, USA), sodium borohydride (95%, Riedel-de Haën, Seelze, Germany). Solutions were prepared in deionized water obtained using a Milli-Q system (Millipore, Direct-Q5, Madrid, Spain). The complete list of reactants can be found in Supplementary Materials S1.

### 2.1. Synthesis of the Sensitive Materials

**Figure 1:**
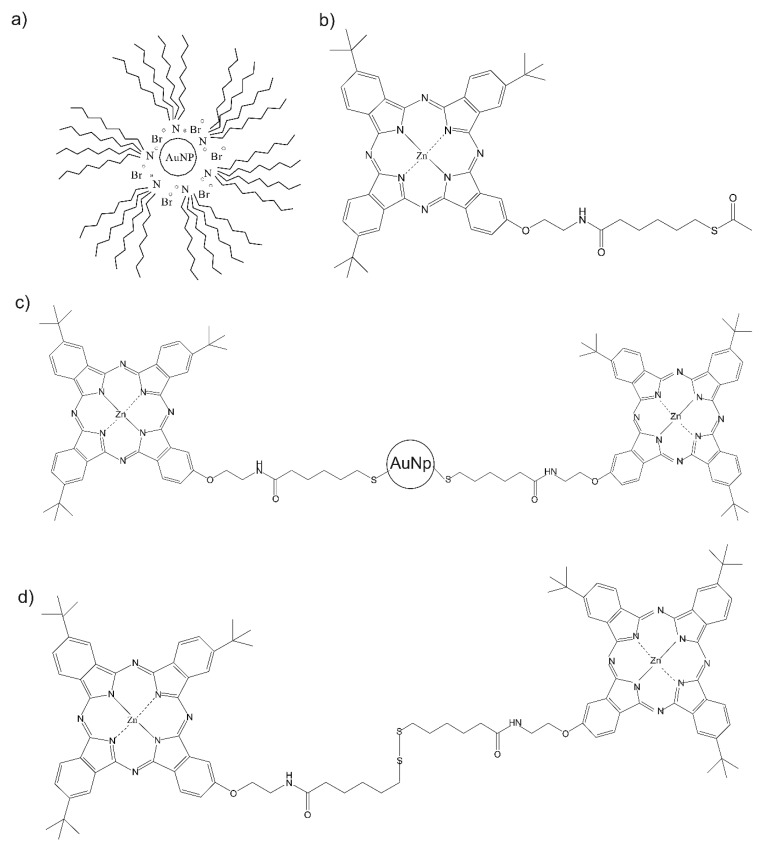
Scheme of the electrocatalytic materials. (**a**) Tetraoctylammonium bromide-capped gold nanoparticles (AuNP^tOcBr^), (**b**) sulfur-substituted zinc phthalocyanine (ZnPc^RS^), (**c**) covalent adduct (AuNP^tOcBr^-S-ZnPc^RS^), (**d**) dimeric sulfur-substituted zinc bisphthalocyanine: (ZnPc^R^-S-ZnPc^R^).

Sensitive materials used in this work are depicted in Figure 1. They were synthesized as follows.

#### 2.1.1. Tetraoctylammonium Bromide-Capped Gold Nanoparticles (AuNP^tOcBr^)

They were synthesized using the Brust method [39]. A water solution of gold tetrachloride was mixed with a toluene solution of tetraoctylammonium bromide. The mixture was stirred until the aqueous phase lost its color, and the organic phase appeared colored. Then, sodium borohydride was added drop by drop to the organic phase until a cherry color was observed. Afterwards, the mixture was stirred under nitrogen atmosphere in darkness. After decantation, gold nanoparticles capped with tetraoctylammonium bromide were obtained as a colloid in toluene.

#### 2.1.2. 6.6′-dithiodihexanoic Acid

It was obtained according to a previously published method [40]. Nine hundred eighty-eight mg (5 mmol) of 6-bromohexanoic acid, 345 mg (2.5 mmol) of K_2_CO_3_ and 0.5 mL of H_2_O were heated at reflux for 20 min. A solution of 1.24 g (5 mmol) of sodium thiosulfate pentahydrate in 3 mL of H_2_O was added and the reaction was allowed to react for 1 h at reflux. Then 1.26 g (5 mmol) of iodine was added and allowed to cool for 30 min. One hundred fifty μL (0.125 mmol) of concentrated H_2_SO_4_ was added, the reaction mixture was diluted in dichloromethane (DCM) and washed with H_2_O, extracting the aqueous phase twice with DCM. The organic phases were dried with Na_2_SO_4_ and the solvent was removed under reduced pressure. The reaction crude was recrystallized from hot toluene to obtain 552 mg of the product (40%), mp 76.5 °C (toluene). ^1^H-RMN (300 MHz, DMSO-*d*_6_, 25 °C): δ = 1.43–1.48 (m, 4H), 1.64–1.73 (m, 8H), 2.2 (t, J = 7.3 Hz, 4H, CH_2_CO), 2.5 (t, J = 7.3 Hz, 4H, CH_2_S), 11.0 (br s, 2H, 2×CO_2_H).^13^C-RMN (75 MHz, DMSO-*d*_6_, 25 °C): δ = 24.1, 27.3, 29.3 (3×CH_2_), 36.1 (CH_2_CO_2_H), 39 (CH_2_S) y 177 (CO_2_H). ν_max_ (KBr)/cm^−1^: 2933, 2856, 1691, 1466, 1434, 1410, 1190 y 922.

#### 2.1.3. Sulfur-Substituted Zinc Phthalocyanine: 2-{2′-[(5′′-Acetylthiopentyloxo)amino]ethoxy}-9(10),16(17),23(24)-tri-tert-butylphalocyaninate Zn(II) (ZnPc^RS^)

It was synthesized following a previously published procedure [21]. The corresponding dimeric structure (ZnPc^R^-S-ZnPc^R^) was synthetized here for the first time using the following method.

#### 2.1.4. Dimeric Sulfur Substituted Zinc Bisphthalocyanine: (ZnPc^R^-S-ZnPc^R^)

As mentioned before, this compound was obtained for the first time in this work. 21 mg (0.024 mmol) of (2-aminoethoxy)-tri-tert-butylphthalocyaninate zinc (II) [16], 3.5 mg (0.012 mmol) of 6,6′-dithiodihexanoic acid and 11.2 mg (0.055 mmol) of dicyclohexylcarbodiimide (DCC) were dissolved in 700 μL of dichloromethane under argon at 0 °C. After 30 min, 1 mg (0.009 mmol) of *N*,*N*-dimethylaminopyridine (DMAP) was added and allowed to react for 3 h. The reaction mixture was diluted with dichloromethane, the organic phase was washed with NH_4_Cl (aq.), NaHCO_3_ (aq.) and H_2_O, dried with Na_2_SO_4_ and the solvent was removed under reduced pressure. The crude was purified by column chromatography (dichloromethane: methanol/99:1) to obtain 14 mg of the compound (60%). ^1^H-RMN (300 MHz, TFA-*d*_1_, 25 °C): δ = 1.47 (m, 12H, 6×CH_2_) 1.68 [br s, 54H, 6x(CH_3_)_3_C], 2.54 (br s, 4H, CH_2_CO), 2.74 (br s, 4H, CH_2_S), 4.16 (br s, 4H, CH_2_N), 4.68 (br s, 4H, CH_2_O), 7.88 (m, 3H, ArH), 8.47 (m, 6H, ArH), 8.96 (m, 2H, ArH) y 9.31–9.48 (m, 13H, ArH). ν_max_ (KBr)/cm^−1^: 3401, 2952, 2855, 1610, 1488, 1461, 1391, 1329, 1255, 1089, 1046 y 748 cm^−1^. UV-Vis (DMF): λmax/nm (log ε): 350 (5.14), 610 (4.84), 676 (5.58). HRMS-MALDI-TOF (dithranol): *m*/*z*: for C_104_H_108_N_18_O_4_S_2_Zn_2_ calcd, 1864.682; found 1864.684 (M^+^).

#### 2.1.5. AuNP^tOcBr^/ZnPc^RS^ and AuNP^tOcBr^/ZnPc^R^-S-ZnPc^R^ Mixtures

The non-covalent mixture of AuNP^tOcBr^/ZnPc^RS^ was prepared from AuNP^tOcBr^ toluene colloid (Abs398 nm = 3, 5 ua) and ZnPc^RS^ (6.5 × 10^−5^ mol∙L^−1^) mixed in a proportion of 2:1 (*v*/*v*). The mixture was kept in the dark until used. A similar method was followed to obtain the mixture AuNP^tOcBr^/ZnPc^R^-S-ZnPc^R^.

#### 2.1.6. AuNP^tOcBr^-S-ZnPc^R^ Covalent Adduct

The covalent adduct (AuNP^tOcBr^-S-ZnPc^R^) was obtained as follows [21]: 4 mL of the phthalocyanine toluene solution (1.3 × 10^−3^ mol∙L^−1^) was mixed with 4 mL of the nanoparticle colloid (Abs398 nm = 3.5 ua) and stirred for 24 h at room temperature, in darkness and under inert atmosphere. Next, the product was added to pentane drop by drop. The precipitate was dissolved in methane and kept overnight at −20 °C. Following centrifugation, the new precipitate of AuNP^tOcBr^-S-ZnPc^R^ was re-suspended in toluene.

### 2.2. Preparation of the Sensors

Sensors were prepared by depositing a layer of the mixtures or of the adduct by spin coating (spin coater model 1H-D7, Micasa Co., Tokyo, Japan). Before deposition, ITO glass substrates were washed with acetone and rinsed twice with deionized water in an ultrasonic bath. Fifty µL of the corresponding material was deposited onto the substrate (1 cm^2^ surface) using 120 s slope and 120 s at 1000 rpm.

The sensing materials and films were characterized by TEM microscopy (JEOL-FS2200 HRP. 200 kV emission) and UV-Vis spectroscopy with a double beam spectrophotometer (UV-2600, Shimadzu, Kyoto, Japan).

### 2.3. Sensing Properties

Cyclic voltammetry was used to characterize the sensing behavior of the chemically modified films. Electrochemical measurements were carried out in a Parstat 2273 (Princeton Applied Research) using a three-electrode cell. The reference electrode was Ag|AgCl/KCl sat. and the counter electrode was a platinum sheet. Modified ITO films were used as working electrodes. The electrochemical responses were analyzed towards catechol 10^−3^ mol∙L^−1^ in phosphate buffer (Na_2_HPO_4_/NaH_2_PO_4_ 0.01 M pH = 7). Cyclic voltammograms were registered from −0.8 to 1.2 V at a scan rate of 0.1 V∙s^−1^. The Limits of detection (LOD) were calculated from peak current responses in voltammograms registered at concentrations from 4 × 10^−6^ to 1.45 × 10^−4^ mol∙L^−1^ following the “3Sd/m” method, where “Sd” is the standard deviation (n = 5) of the signal registered in the buffer, and “m” is the slope of the calibration curve. The influence of the potential sweep rate was studied in catechol 10^−4^ mol∙L^−1^ changing the scan rates from 0.01 to 1.0 V∙s^−1^.

## 3. Results and Discussion

**Figure 2:**
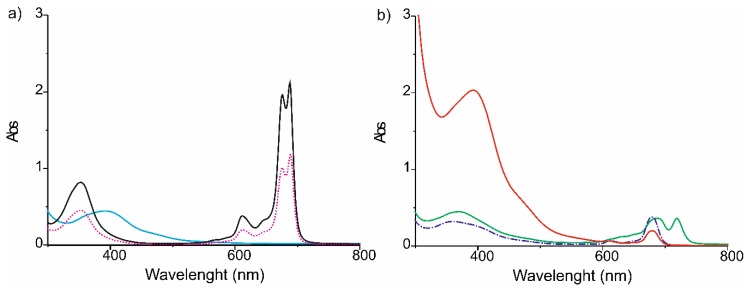
UV-Vis absorption spectra of (**a**) AuNP^tOcBr^ (blue —), ZnPc^RS^ (pink ······), ZnPc^R^-S-ZnPc^R^ (black—) and of (**b**) the mixture AuNP^tOcBr^/ZnPc^RS^ (purple ∙–∙–∙), the mixture AuNP^tOcBr^/ZnPc^R^-S-ZnPc^R^ (green —) and the covalent adduct AuNP^tOcBr^-S-ZnPc^R^ (red —), in toluene as solvent.

The UV-Vis spectra of the individual sensing materials are shown in Figure 2a. The electronic absorption spectrum of the AuNP^tOcBr^ colloid was dominated by an intense peak at 398 nm produced by the plasmon resonance, accompanied by a small shoulder at 485 nm. The sharpness of the peak at 398 nm reflected a homogeneous distribution of the NPs size. The colloid diluted 1:10, showed the same features as the undiluted colloid, confirming the lack of aggregation. UV-Vis spectra of the ZnPc^RS^ toluene solutions showed the expected Q bands at 689 nm and at 675 nm which are usually observed in unsymmetrical phthalocyanines. The spectrum also exhibited an intense Soret band at 353 nm and a small vibronic band at 618 nm. The spectrum of the dimeric phthalocyanine was similar to the one observed in the monomeric form. The only differences were found in the intensity of the Q and Soret bands which were more intense in the dimeric compound due to the presence of two phthalocyanine rings. The UV-Vis spectra of the mixtures and of the adduct are presented in Figure 2b. The spectrum of the AuNP^tOcBr^/ZnPc^RS^ mixture showed bands associated with each one of the components, although changes in the intensities and positions of the peaks with respect to those observed in the spectra of the individual components were observed: The Q band of the phthalocyanine appeared at 679 nm. Due to its broadness, the splitting was no longer observed. Furthermore, the Soret band increased its intensity with respect to the Q band, and appeared at 359 nm, overlapping with the plasmonic band of the nanoparticle.

The UV-Vis spectrum of the covalent adduct AuNP^tOcBr^-S-ZnPc^R^ showed the same features as shown by the mixture. However, a clear increase in the intensity of the band at 393 nm produced by the overlapping of the phthalocyanine Soret band and the band of the AuNP^tOcBr^ plasmon band was observed. This effect was consistent with a covalent interaction between the phthalocyanine and the nanoparticle that caused the modification of the π-π transition.

The mixture with the dimer AuNP^tOcBr^/ZnPc^R^-S-ZnPc^R^ showed two broad Q bands. The first broadband at 685 nm is produced by the substituted Pc ring similar to that observed in the monomeric species. The splitting observed in the monomer cannot be observed due to the broadness of the band. The second band at 719 nm is typical of the formation of J aggregates due to the interaction between the two Pc rings. The Soret band appears overlapped with the band corresponding to the plasmon resonance of the nanoparticles. Obviously, a covalent adduct could not be obtained by reaction of the dimer and the AuNPs because the covalent bond was not accessible.

**Figure 3:**
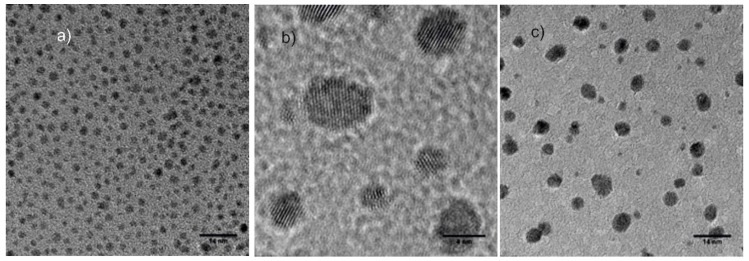
TEM images of (**a**) AuNP^tOcBr^, (**b**) mixture AuNP^tOcBr^/ZnPc^RS^, and (**c**) adduct AuNP^tOcBr^-S-ZnPc^R^.

According to TEM images (Figure 3), the estimated core diameter of the AuNP^tOcBr^ was 2–3 nm. The images of the mixtures AuNP^tOcBr^/ZnPc^RS^ and of the adduct AuNP^tOcBr^-S-ZnPc^R^ showed nanoparticles with sizes ranging from 2 to 5 nm, with an average value of 4 nm. The images also revealed the existence of a light halo surrounding the nanoparticles, which was due to the phthalocyanines located around nanoparticles. The thickness of the halo was smaller in the case of the mixtures AuNP^tOcBr^/ZnPc^RS^ and AuNP^tOcBr^/ZnPc^R^-S-ZnPc^R^ films and could only be observed at higher magnifications.

ITO glasses were modified with spin-coated films of the AuNP^tOcBr^/ZnPc^RS^, AuNP^tOcBr^/ZnPc^R^-S-ZnPc^R^ mixtures and of the AuNP^tOcBr^-S-ZnPc^R^ adduct. Their sensing properties towards catechol were analyzed using cyclic voltammetry. The electrochemical responses of a bare ITO and films prepared from individual components AuNP^tOcBr^, ZnPc^RS^, and ZnPc^R^-S-ZnPc^R^ were also analyzed for comparison purposes.

**Figure 4:**
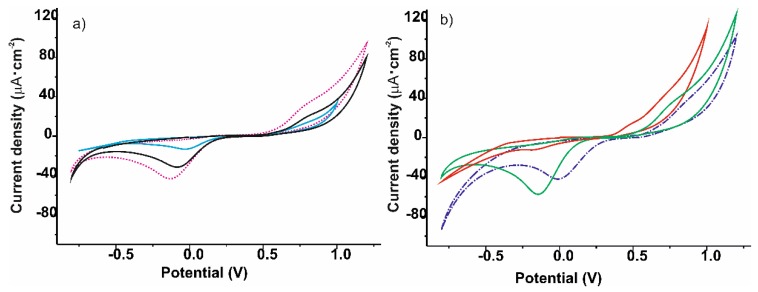
Cyclic voltammograms registered in catechol 10^−3^ mol∙L^−1^ (0.01 M phosphate buffer as electrolyte using (**a**) AuNP^tOcBr^ (blue —), ZnPc^RS^ (pink ······), ZnPc^R^-S-ZnPc^R^ (black —) and of (**b**) the mixture AuNP^tOcBr^/ZnPc^RS^ (purple ∙–∙–∙), the mixture AuNP^tOcBr^/ZnPc^R^-S-ZnPc^R^ (green —) and the covalent adduct AuNP^tOcBr^-S-ZnPc^R^ (red —), Scan rate 100 mV∙s^−1^.

Voltammetric responses towards a 10^−3^ mol∙L^−1^ catechol solution (in 0.01 M phosphate buffer as electrolyte pH = 7) are shown in Figure 4. As a general rule, responses were characterized by an anodic peak at positive potentials (produced by the oxidation of catechol to 1, 2 benzoquinone) and a cathodic peak at ca. −0.25 V produced by the corresponding reduction of the benzoquinone. However, important differences were caused by the modification of the electrode.

When a bare ITO electrode was immersed in catechol, peaks were quite weak. A small increase in the intensity of the peaks was observed when the ITO glass was coated with AuNP^tOcBr^. In contrast, ZnPc^RS^ coated ITO glass produced an increase in the intensity of the anodic wave (from 3 µA in ITO to 30 µA in films coated with ZnPc^RS^). The cathodic peak also increased from −7 µA to −45 µA. The observed increase proved the electrocatalytic properties of the zinc phthalocyanine derivative.

Voltammograms obtained using electrodes modified with the AuNP^tOcBr^/ZnPc^RS^ mixture also showed the expected anodic and cathodic waves. The anodic peak was almost identical to that obtained with ZnPc^RS^ alone, indicating that the influence on the electrocatalytic behavior of the AuNP^tOcBr^ present in the mixture was almost negligible. In contrast, the position of the cathodic peak shifted to lower potentials and the mixture of compounds seemed to show a stronger electrocatalytic effect than the components separately. The mixture of gold nanoparticles with the dimeric species AuNP^tOcBr^/ZnPc^R^-S-ZnPc^R^, produced a higher increase in the intensity of the cathodic wave than the mixture of the nanoparticle with the monomeric phthalocyanine. This could be due to the stronger interaction between the phthalocyanine rings and the gold NPs.

Responses observed using electrodes modified with the covalent adduct AuNP^tOcBr^-S-ZnPc^R^ differed from those obtained with the AuNP^tOcBr^/ZnPc^RS^ mixture. The main difference was observed in the anodic peak that showed an important shift to lower potentials. In contrast, the electrocatalytic effect disappeared completely in the cathodic peak.

The important differences between the mixture and the adduct confirm the importance of the nature of the interaction between the phthalocyanine and the gold nanoparticle in the electrocatalytic mechanism.

In order to further analyze the effect of the modifiers on the dynamic character of the electrochemical process, voltammograms were registered at different scan rates (from 0.01 to 1.0 V∙s^−1^). Experiments were carried out in catechol 10^−4^ mol∙L^−1^ (in phosphate buffer 0.01 M, pH = 7). In all cases, the intensity of the peaks increased with the scan rate. Simultaneously, cathodic peaks shifted to more negative potentials while anodic peaks shifted to more positive potentials.

**Figure 5:**
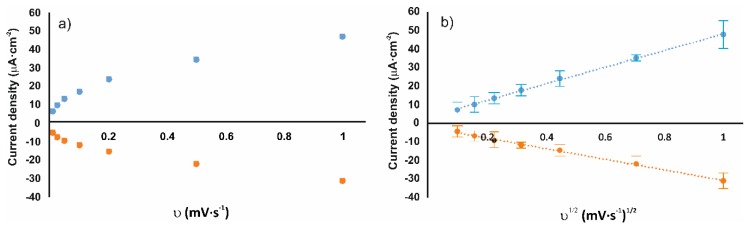
Analysis of the dynamic behavior of the sensor based on the mixture AuNP^tOcBr^/ZnPc^R^-S-ZnPc^R^ (**a**) Laviron model, graphical relationship between the current peak density and the sweep rate (*ν*), (**b**) Randles–Sevcik model peak current density varies linearly with the square root of the scan rate (*ν*^1/2^).

According to the literature, when the peak current varies linearly with the sweep rate (*ν*), the transfer of the electrons from the analyte to the electrode is the limiting step of the process. If the peak current varies linearly with the square root of the scan rate (*ν*^1/2^), the electrode reaction is controlled by diffusion. Figure 5 shows the analysis of the dynamic behavior of the sensor based on the mixture AuNPtOcBr/ZnPcR-S-ZnPcR. Figure 5a shows the relationship between the current density and the sweep rate (*ν*), according to the Laviron model (Equation (1)) and Figure 5b shows the relationship between the current density and the square root of the scan rate (*ν*^1/2^) according to the Randles–Sevcik model (Equation (2)). Slopes and correlation coefficients for all the sensors are collected in Table 1.

(1) $$Ic=\frac{n^{2}F^{2}\nu \Gamma A}{4RT}$$

(2) $$Ic=0.446FA\sqrt{\frac{FD\nu }{RT}}\left[C\right]$$

where *I_c_* is the peak current, *n* is the number of electrons involved in the process, *F* is the Faraday’s constant, *ν* is the scan rate (expressed in V∙s^−1^), *Γ* is the surface coverage of the electrode reaction substance (mol cm^−2^), *A* is the electrode area (cm^2^), *R* is the ideal gas constant (8.314 J·mol^−1^·K^−1^), *T* is the temperature (298 K). *D* is the diffusion coefficient, [*C*] the bulk concentration of species *C* in the solution.

As observed in Figure 5 and Table 1, correlation coefficients R^2^ show that both models, Laviron and Randless–Sevcik could explain the dynamic behavior of the sensors. This is quite common in chemically modified electrodes immersed in electroactive solutions. However, the fitting is clearly linear in the diffusion-controlled model.

**Figure 6:**
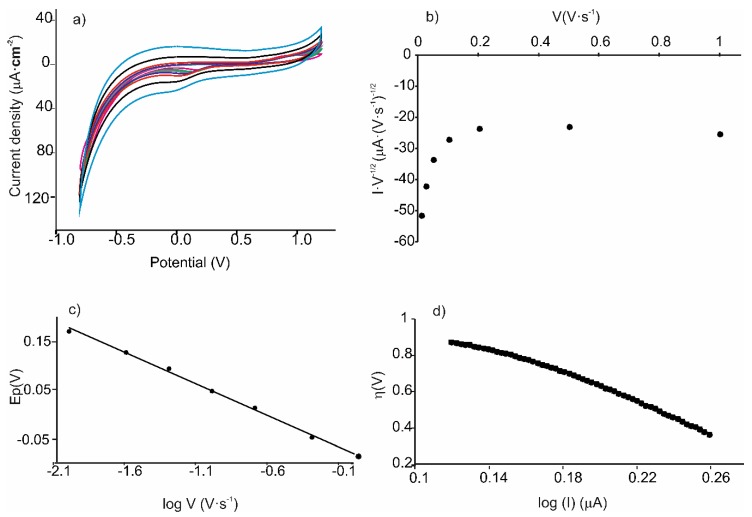
Analysis of the electron transfer mechanism. (**a**) Cyclic voltammograms of AuNP^tOcBr^/ZnPc^RS^ in catechol 10^−4^ mol∙L^−1^ registered at increasing scan rates from 0.01 to 1 V∙s^−1^, (**b**) representation of I·*ν*^−1/2^ vs. scan rate for the cathodic pea, (**c**) variation of peak potentials vs. the logarithm of the scan rates. (**d**) Representation of Tafel plot: overpotential η vs log (I) in cathodic peak.

In order to further analyze the nature of the limiting step of the electrode reaction, the relationship between I/*ν*^1/2^ vs. *ν* was analyzed. If this relationship is linear, the mechanism that controls the redox process is the charge transfer of the adsorbate. On the contrary, when the current function I/*ν*^1/2^ is independent of the scan rate, the predominant mechanism is diffusion. In this case, all sensors showed a combination of both mechanisms: At low scan rates (lower than 0.20 V∙s^−1^), the charge transfer predominated. At scan rates over 0.20 V∙s^−1^, the process was limited by diffusion (Figure 6).

At scan rates lower than 0.20 V∙s^−1^, where the charge transfer is the limiting step, the charge transfer coefficient α can be calculated from the slope of the Laviron equation (Equation (3)). This coefficient is related to the efficiency of the electron transfer between the electrode and the surface-confined redox couple [36],

(3) $$E_{c}=E^{0}-\frac{2.3RT}{(\alpha_{c})nF}\log\upsilon$$

where *E_c_* is the cathodic peak potential, *E*^0^ is a constant that includes the formal standard potential, *R* is the ideal gas constant (8.314 J·mol^−1^·K^−1^), *T* is the temperature (298 K), *α_c_* is the charge transfer coefficient, *n* is the number of electrons involved in the process, *F* is the Faraday’s constant and *ν* is the scan rate (expressed in V∙s^−1^).

Our results showed that the slope of the *E_c_* vs. log *ν* gave αn values between 0.43 and 0.45 (Table 2).

In order to obtain information about the efficiency of the catalyst and the rate-determining step, representation of log *I* (µA) vs. the overpotential, *η* (V), Tafel plot was used to calculate values thanks to the simplified Butler–Volmer equation (Equation (4).

(4) $$LogI=\log I_{0}-\frac{\alpha F}{2.3RT}\eta$$

The *α* values obtained can be substituted in Laviron’s equation to calculate the number of electrons implicated in the redox process. All these values are listed in Table 2. Calculations indicate a two-electron redox reaction of catechol at all three electrodes.

Similar calculations were carried out using the anodic peak (Equation (5) and (6)) where *E_a_* is the anodic peak potential.

(5) $$E_{a}=E^{0}-\frac{2.3RT}{(1-\alpha_{})nF}\log\upsilon$$

(6) $$LogI=\log I_{0}-\frac{(1-\alpha )F}{2.3RT}\eta$$

As observed in the table, the charge transfer coefficient *α*, showed different values in the AuNP^tOcBr^/ZnPc^RS^ composite than in the AuNP^tOcBr^-S-ZnPc^R^ adduct, confirming the different mechanism of the reduction process. It is noteworthy that the behavior of the mixture containing the dimer (AuNP^tOcBr^/ZnPc^R^-S-ZnPc^R^) where the thiol group is protected coincided with that of the mixture AuNP^tOcBr^/ZnPc^RS^. This confirms that the interaction between the phthalocyanine and the nanoparticle did not occur through thiol bonds.

**Figure 7:**
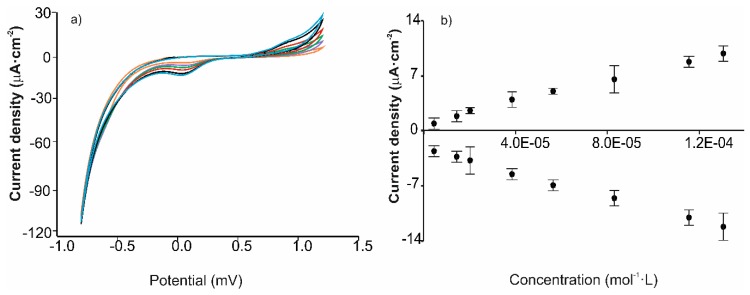
Analysis of limits of detection. (**a**) Voltammetric response of the AuNP^tOcBr^/ZnPc^AcS^ sensor to increasing concentrations of catechol (from 4 × 10^−6^ to 1.40 × 10^−4^ mol∙L^−1^ in phosphate buffer), (**b**) calibration curves calculated in both the anodic and the cathodic peaks.

The limits of detection (LOD) were calculated from voltammograms registered in solutions with increasing concentrations of catechol (from 4.0 × 10^−6^ to 1.40 × 10^−4^ mol∙L^−1^). Experiments were replicated three times for each sensor. As expected, the intensity of the peaks increased with the concentration and the responses were linear in the studied range (Figure 7). Calibration curves were constructed by representing *I_a_* (or *I_c_)* vs. catechol concentration. Sensitivity and LODs were calculated from those plots. The results are shown in Table 3. As expected, and according to the α parameters obtained from the experiments carried out at different sweep rates, the presence or absence of a covalent bond influenced the sensitivities and the LODs.

According to Table 3, LODs obtained from the cathodic curves were lower in sensors modified with the mixtures (0.9 × 10^−6^ M for AuNP^tOcBr^/ZnPc^RS^ and 1.2 × 10^−6^ M AuNP^tOcBr^/ZnPc^R^-S-ZnPc^R^). These values were quite similar to those obtained with the ZnPc^R^ alone indicating a weak electrocatalytic effect. The AuNP^tOcBr^-S-ZnPc^R^ covalent adduct did not show any electrocatalytic effect in the cathodic process. These values are similar to those obtained with other sensors modified with nanoparticles or phthalocyanines separately [2,10,36,38,41,42].

Results were completely different in the anodic wave. LODs calculated from the anodic peaks, showed that the sensor modified with the covalent adduct gave the lowest LOD values (1.38 × 10^−7^ mol∙L^−1^), confirming the strong influence of the covalent bond in the mechanism of catechol oxidation. This result indicates that the covalent interaction facilitated the electron transfer during oxidation and that the nature of the interaction between both components (weak bond in the mixture or covalent bond in the adduct) modulates the catalytic activity.

## 4. Conclusions

New voltammetric sensors based on combinations of gold nanoparticles and sulfur substituted zinc phthalocyanines have been developed and used as electrochemical sensors for the detection of catechol. The electron transfer process, as well as the existence of synergistic effects between both components in the absence and presence of covalent links has been analyzed.

It has been demonstrated that the electrocatalytic properties and the kinetic parameters depend on the type of interaction between both components. The AuNP^tOcBr^/ZnPc^RS^ and the AuNP^tOcBr^/ZnPc^R-^S-ZnPc^R^ mixtures enhance the electron transfer rate of the catechol reduction. Both modifiers showed similar LODs of 10^−6^ mol∙L^−1^. As in the dimeric phthalocyanine the sulfur group is blocked, it can be inferred that the sulfur group does not play a role in the electrocatalytic process. In turn, the AuNP^tOcBr^-S-ZnPc^R^ covalent adduct facilitates the oxidation of catechol, showing an enhanced charge transfer rate, and an LOD of 10^−7^ mol∙L^−1^.

Under the light of these results, combining covalently nanoparticles and phthalocyanines can be considered a good strategy to improve the charge transfer rate and the limits of detection of catechol. Future works should be dedicated to analyzing the effect of the interaction between electrocatalytic materials in other systems different than the nanoparticle-phthalocyanine system.

## Figures and Tables

**Table 1:** Relationship between the intensity of the peaks and the scan rate in sensors immersed in 10^−4^ mol·L^−1^ catechol. (Results shown correspond to the average values obtained from three different experiments).

Cathodic Wave at ca. –0.15 V						
	Laviron Model: *I*= f(*ν*), *I_c_* (µA∙cm^−2^) vs. *ν* (V/s)			Randless–Sevcik Model *I* = f (sqrt(*ν*)) *I_c_* (µA∙cm^−2^) vs. *ν*^1/2^ (V/s)^1/2^		
**Sensor**	**Slope**	**Intercept**	**R^2^**	**Slope**	**Intercept**	**R^2^**
AuNP^tOcBr^/ZnPc^RS^	−19.32	−6.11	0.993	−21.31	−2.32	0.973
AuNP^tOcBr^/ZnPc^R^-S-ZnPc^R^	−24.78	−7.59	0.953	−28.26	−2.34	0.997
AuNP^tOcBr^-S-ZnPc^R^	−54.46	−5.33	0.915	−31.65	−1.64	0.982
**Anodic wave at ca. 0.8 V**						
	***I_c_* (µA∙cm^−2^) vs. *ν* (V/s)**			***I_c_* (µA∙cm^−2^) vs. *ν*^1/2^ (V/s)^1/2^**		
**Sensor**	**Slope**	**Intercept**	**R^2^**	**Slope**	**Intercept**	**R^2^**
AuNP^tOcBr^/ZnPc^RS^	42.12	6.34	0.980	37.20	−0.06	0.962
AuNP^tOcBr^/ZnPc^R^-S-ZnPc^R^	38.85	11.71	0.935	44.76	3.30	0.998
AuNP^tOcBr^-S-ZnPc^R^	210.91	10.45	0.934	92.17	1.82	0.987

**Table 2:** Relationship with scan rate in sensors immersed in catechol 10^−4^ mol∙L^−1^.

Cathodic Peak									
	*I_c_/ν*^1/2^ vs. *ν*		Log *I* vs. *ƞ*			*E_c_* vs. log *ν*			
**Sensor**	**Slope**	**R^2^**	**Slope**	R^2^	***α***	**Slope**	**R^2^**	***α*n**	**n**
AuNP^tOcBr^/ZnPc^AcS^	252.39	0.882	3.73	0.986	0.28	0.130	0.997	0.452	2.05
AuNP^tOcBr^/ZnPc^R^-S-ZnPc^R^	177.68	0.947	4.43	0.979	0.26	−0.105	0.997	0.562	2.14
AuNP^tOcBr^-S-ZnPc^R^	102.29	0.958	2.912	0.997	0.17	0.136	0.990	0.434	2.43
**Anodic Peak**									
	***I_c_/ν*^1/2^ vs. *ν***		**Log *I* vs. *ƞ***			***E_c_* vs. log *ν***			
**Sensor**	**Slope**	**R^2^**	**Slope**	**R^2^**	***α***	**Slope**	**R^2^**	***α*n**	**n**
AuNP^tOcBr^/ZnPc^AcS^	−244.14	0.872	2.34	0.998	0.76	0.189	0.953	0.313	2.28
AuNP^tOcBr^/ZnPc^R^-S-ZnPc^R^	−153.15	0.888	3.88	0.999	0.77	0.113	0.985	0.522	2.28
AuNP^tOcBr^-S-ZnPc^R^	−137.50	0.964	1.579	0.999	0.90	0.331	0.992	0.165	1.76

**Table 3:** Sensitivity, limit of detection (LD) and correlation coefficient (R^2^).

	Sensor	Sensitivity (µA∙cm^−2^/mol∙L^−1^)	LOD (× 10^−6^ mol∙L^−1^)	R^2^
Cathodic peak	AuNP^tOcBr^	−23,747	4.0	0.992
	ZnPc^RS^	−87,223	2.0	0.987
	AuNP^tOcBr^/ZnPc^RS^	−76,350	0.9	0.997
	AuNP^tOcBr^/ZnPc^R^-S-ZnPc^R^	−99,039	1.2	0.989
	AuNP^tOcBr^-S-ZnPc^R^	−32,419	8.3	0.989
Anodic peak	AuNP^tOcBr^	10,539	4.4	0.992
	ZnPc^RS^	28,343	2.9	0.985
	AuNP^tOcBr^/ZnPc^RS^	68,170	2.2	0.994
	AuNP^tOcBr^/ZnPc^R^-S-ZnPc^R^	44,337	2.07	0.981
	AuNP^tOcBr^-S-ZnPc^R^	45,498	0.13	0.993

## Supplementary Materials

The following are available online at https://www.mdpi.com/2079-4991/9/11/1506/s1.
