# Supplementary material for: Electrochemical Sensors Modified with Combinations of Sulfur Containing Phthalocyanines and Capped Gold Nanoparticles: A Study of the Influence of the Nature of the Interaction between Sensing Materials
Source: Nanomaterials (Basel). 2019 Oct 23;9(11):1506. doi: 10.3390/nano9111506 (PMC6915348; doi:10.3390/nano9111506)
Supplement: Supplementary file 1 [file nanomaterials-09-01506-s001.pdf]

Supplementary material

Electrochemical Sensors Modified with Combinations of Sulfur Containing Phthalocyanines and Capped Gold Nanoparticles: A Study of the Influence of the Nature of the Interaction between Sensing Materials

Ana Isabel Ruiz-Carmuega [1], Celia Garcia-Hernandez [1,2], Javier Ortiz [3], Cristina Garcia-Cabezon [1,2], Fernando Martin-Pedrosa [1,2], Angela Sastre-Santos [3], Miguel Angel Rodríguez-Perez [2] and Maria Luz Rodriguez-Mendez [1,2,*]

1 Group UVASENS, Escuela de Ingenierías Industriales, Universidad de Valladolid, Paseo del Cauce, 59, 47011 Valladolid, Spain; anaisabel.ruiz@uva.es (A.I.R.-C.); celiagarciahernandez@gmail.com (C.G.-H.); crigar@eii.uva.es (C.G.-C.); fmp@eii.uva.es (F.M.-P.)
2 BioecoUVA Research Institute, Universidad de Valladolid, 47011 Valladolid, Spain; marrod@fmc.uva.es
3 Área de Química Orgánica, Instituto de Bioingeniería, Universidad Miguel Hernández de Elche, 03202 Elche, Spain; jortiz@umh.es (J.O.); asastre@umh.es (A.S.-S.)
* Correspondence: mluz@eii.uva.es; Tel.: +34-983-423-540

Contents

Sources of the reagents and substrates used in this work ….............................. S1

1. Reagents to prepare Tetraoctylammonium bromide-capped gold nanoparticles ($AuNP^{tOcBr}$):

$HAuCl_4 \cdot xH_2O$ (12325, 99.9%, min. 49% Au, Alfa Aesar), tetraoctylammonium bromide (294136, 98%, Aldrich), sodium borohydride (62861, 95%, Riedel-de Haën). Solutions were prepared in deionized water obtained using a Milli-Q system (Millipore, Direct-Q5).

2. Reagents to prepare 6.6'-dithiodihexanoic acid

6-Bromohexanoic acid (150452, 98%) was purchased from Aldrich. Potassium carbonate (ACS reagent, ≥99%), Sodium thiosulfate pentahydrate (ACS reagent, 99.5%), iodine (15600660, 99.8%), concentrated sulphuric acid (ACS reagent, 95.0-98.0%), dichloromethane (HPLC Grade), sodium sulfate (ACS reagent, ≥99.0%, anhydrous, granular), toluene (anhydrous, 99.8%).

3. Reagents to prepare Sulfur Substituted zinc phthalocyanine ($ZnPc^{RS}$):

6-Bromohexanoic acid (150452, 98%), 2-chloro-4,6-dimethoxy-1,3,5-triazine (97%) (CDMT), 4-methylmorpholine (ReagentPlus®, 99%) (NMM) were purchased from Aldrich. Tetrahydrofuran (anhydrous, ≥99.9%), ethyl acetate (anhydrous, 99.8%) sodium hydrogen carbonate (ACS reagent, ≥99.7%), magnesium sulfate (anhydrous, reagent grade, ≥99.5%), dichloromethane (HPLC Grade).

4. Reagents to prepare Dimeric Sulfur Substituted zinc bisphthalocyanine: ($ZnPc^{R}$-S-$ZnPc^{R}$):

Dicyclohexylcarbodiimide (DCC, 36650, 99%) and 4-dimethylaminopyridine (DMAP, 39405, 98%) were purchased from Honeywell Fluka. dichloromethane (HPLC Grade), sodium sulfate (ACS reagent, ≥99.0%, anhydrous), sodium hydrogen carbonate (ACS reagent, ≥99.7%), ammonium chloride (ACS reagent, ≥99.5%).
